# The association of physical activity and cardiorespiratory fitness with β-cell dysfunction, insulin resistance, and diabetes among adults in north-western Tanzania: A cross-sectional study

**DOI:** 10.3389/fendo.2022.885988

**Published:** 2022-08-03

**Authors:** Brenda Kitilya, Robert Peck, John Changalucha, Kidola Jeremiah, Bazil B. Kavishe, Henrik Friis, Suzanne Filteau, Rikke Krogh-Madsen, Soren Brage, Daniel Faurholt-Jepsen, Mette F. Olsen, George PrayGod

**Affiliations:** ^1^ Mwanza Research Centre, National Institute for Medical Research, Mwanza, Tanzania; ^2^ Department of Internal Medicine and Pediatrics, Weill Bugando School of Medicine, Mwanza, Tanzania; ^3^ Department of Global Health, Weill Cornell Medicine, New York, NY, United States; ^4^ Department of Nutrition, Exercise and Sports, University of Copenhagen, Copenhagen, Denmark; ^5^ Department of Population Health, London School of Hygiene and Tropical Medicine, London, United Kingdom; ^6^ Centre for Physical Activity Research, Rigshospitalet, University of Copenhagen, Copenhagen, Denmark; ^7^ Department of Infectious Diseases, Copenhagen University Hospital, Copenhagen, Denmark; ^8^ Medical Research Council (MRC) Epidemiology Unit, University of Cambridge, Cambridge, United Kingdom; ^9^ Department of Infectious Diseases, Rigshospitalet, Copenhagen, Denmark

**Keywords:** physical activity energy expenditure, sleeping heart rate, VO_2_max, insulinogenic index, HOMA-β, overall insulin release, HOMA-IR, Sub-Saharan Africa

## Abstract

**Introduction:**

Research on the associations of physical activity and cardiorespiratory fitness with β-cell dysfunction and insulin resistance among adults in Sub-Saharan Africa (SSA) is limited. We assessed the association of physical activity and cardiorespiratory fitness with β-cell function, insulin resistance and diabetes among people living with HIV (PLWH) ART-naïve and HIV-uninfected Tanzanian adults.

**Method:**

In a cross-sectional study, we collected data on socio-demography, anthropometry, fat mass and fat free mass and C-reactive protein. Data on glucose and insulin collected during an oral glucose tolerance test were used to assess β-cell dysfunction (defined as insulinogenic index <0.71 (mU/L)/(mmol/L), HOMA-β index <38.3 (mU/L)/(mmol/L), and overall insulin release index <33.3 (mU/L)/(mmol/L)), oral disposition index <0.16 (mU/L)/(mg/dL)(mU/L)^-1^, insulin resistance (HOMA-IR index >1.9 (mU/L)/(mmol/L) and Matsuda index <7.2 (mU/L)/(mmol/L), prediabetes and diabetes which were the dependent variables. Physical activity energy expenditure (PAEE), sleeping heart rate (SHR), and maximum uptake of oxygen during exercise (VO_2_ max) were the independent variables and were assessed using a combined heart rate and accelerometer monitor. Logistic regressions were used to assess the associations.

**Results:**

Of 391 participants, 272 were PLWH and 119 HIV-uninfected. The mean age was 39 ( ± 10.5) years and 60% (n=235) were females. Compared to lower tertile, middle tertile of PAEE was associated with lower odds of abnormal insulinogenic index (OR=0.48, 95%CI: 0.27, 0.82). A 5 kj/kg/day increment of PAEE was associated with lower odds of abnormal HOMA-IR (OR=0.91, 95%CI: 0.84, 0.98), and reduced risk of pre-diabetes (RRR=0.98, 95%CI: 0.96, 0.99) and diabetes (RRR=0.92, 95%CI: 0.88, 0.96). An increment of 5 beats per min of SHR was associated with higher risk of diabetes (RRR=1.06, 95%CI: 1.01, 1.11). An increase of 5 mLO_2_/kg/min of VO_2_ max was associated with lower risk of pre-diabetes (RRR=0.91, 95%CI: 0.86, 0.97), but not diabetes. HIV status did not modify any of these associations (interaction, p>0.05).

**Conclusion:**

Among Tanzanian adults PLWH and HIV-uninfected individuals, low physical activity was associated with β-cell dysfunction, insulin resistance and diabetes. Research is needed to assess if physical activity interventions can improve β-cell function and insulin sensitivity to reduce risk of diabetes and delay progression of diabetes in SSA.

## Introduction

Globally, 8.5% of adults aged ≥18 years had diabetes in 2014 (1). Diabetes was responsible for deaths of 1.5 million people in 2019 and the World Health Organization (WHO) projects that it will be the leading cause of mortality by 2040 and that low-and middle-income countries, including those in Sub-Saharan Africa (SSA), will be affected disproportionately (1).

Diabetes develops due to β-cell dysfunction, insulin resistance, or a combination of these (2). In SSA, the increasing diabetes burden is partly driven by insulin resistance from overweight particularly seen in urban settings, associated with intake of high-calorie low-fibre diets, low levels of physical activity (3, 4), and from infections like HIV (5, 6). However, reduced insulin secretion due to under nutrition could also contribute to diabetes (7, 8).

Physical activity and cardiorespiratory fitness are associated with improved glucose metabolism and reduce the risk of insulin resistance and diabetes (9, 10). Specifically, studies have reported physical activity improves β-cell function by increasing either β-cell mass or insulin secretion (11). However, data showing these associations come from high-income countries and are mostly based on reported rather than objective physical activity data (12). Thus, objectively collected data are needed to advocate for the need for physical activity as a strategy to prevent diabetes in SSA. In addition, since both β-cell dysfunction and insulin resistance contribute to the pathogenesis of diabetes (13), it would be insightful to investigate if physical activity is associated with both improved β-cell function and insulin sensitivity in the African populations.

Traditionally, objectively, physical activity has been assessed using doubly labelled water (a gold standard for assessing free living physical activity energy expenditure), heart rate monitors and accelerometers (14). While doubly labelled water may be expensive, accelerometers and heart rate monitors have gained prominence because they are cheaper yet accurate (15). However, accelerometers are unable to measure individual heart rate for estimation of physical activity energy expenditure (16, 17). Similarly, cardiorespiratory fitness has been assessed using maximum uptake of oxygen during exercise (VO_2_ max) with walk test or step test (18). More recently, a combined accelerometer and heart rate monitor has been found to be accurate in producing both physical activity energy expenditure and cardiorespiratory fitness data (19–22). The method is easier to apply and has been previously applied in Africa including among people living with HIV (23, 24).

We aimed to assess the associations between physical activity energy expenditure (PAEE), and cardiorespiratory fitness with β-cell dysfunction, insulin resistance, prediabetes and diabetes among Tanzanian antiretroviral therapy (ART)-naïve people living with HIV (PLWH) and HIV-uninfected adults.

## Methods

### Study design and setting

A cross-sectional study was conducted from February 2017 to February 2018 in Mwanza, Tanzania, where the prevalence of HIV infection was 7.2% (25). This study was embedded within the Chronic Infections, Co-morbidities and Diabetes in Africa (CICADA) cohort (5), a prospective study investigating diabetes and HIV registered at http://clinicaltrials.gov as NCT03106480.

### Sample size

Based on previous studies we anticipated that high level of physical activity would reduce the odds of having diabetes by 60% (5). Assuming that 20% of the participants who are not physically active will have diabetes (26) and the ratio of participants who are physically inactive to those who are active would be around 0.4 (5), we would need 248 participants who are physically active (defined as those in the middle and upper tertiles of PAEE) and 99 physically inactive participants to demonstrate an odds ratio of 0.4 or lower for the association between physical activity and diabetes with 80% power at 0.05 significant level (27).

### Recruitment of participants

PLWH and HIV-uninfected participants from the CICADA cohort were invited to participate in this study. The study inclusion criteria for PLWH were ≥18 years, newly diagnosed with HIV, willing to start ART, not pregnant, and residing in Mwanza. PLWH who have not initiated ART were recruited and assessed before they started ART at local ART clinics without compromising the WHO approved test and treat policy (28). The recruitment of HIV-uninfected adults was conducted from the same communities as the PLWH and included half as many HIV-uninfected adults as PLWH. With support from community leaders, we randomly selected three households from which an eligible individual who met the inclusion criteria (i.e. not seriously sick, not pregnant, and not having any HIV clinical symptoms) was identified and invited to the study. Participants were then tested to confirm their HIV-status and if they were found HIV-infected, they were referred for treatment to the ART clinic.

### Data collection procedures

Demography and socio-economic status data were collected using electronic structured questionnaires in a CSpro data capturing system (CSPro 6.3, Census bureau, USA).

Anthropometric data were collected in triplicate using standardized methods and the median was used for analysis. With minimal clothing and while barefoot, participant’s body weight was measured to the nearest 0.1 kg using a digital scale (Seca, Hamburg, Germany). Height was measured to the nearest 0.1 cm using a wall-fitted stadiometer (Seca, Hamburg, Germany) and waist circumference was measured using a non-stretchable tape measure. Based on the WHO cut-off points, body mass index (BMI) was calculated as weight (kg)/height (m)^2^ and classified as underweight (<18.5 kg/m^2^), normal weight (18.5-24.99 kg/m^2^), overweight (>24.99-<30.0 kg/m^2^) and obese (≥30kg/m^2^). Waist circumference was defined as normal (women: ≤88 cm; men: ≤102 cm) and abdominal obesity (women: >88 cm; men: >102 cm) (29).

PAEE ((kj/kg/day) was a proxy measure of physical activity, whereas sleeping heart rate (SHR) (beats/min) and VO_2_max were the proxy measures of cardiorespiratory fitness (30, 31). Heart rate and acceleration were assessed in a free-living environment for five days using a combined heart rate and accelerometer monitor (Actiheart, Camtech, Cambridge, UK) (17, 19). and used to derive PAEE and SHR. A minimum of three full days of monitoring was needed to provide valid results. The device was fitted on a participant using two electrocardiogram (ECG) electrodes placed one on the upper left side of the chest and another one placed laterally on top of the chest.

Heart rate and activity data from free-living were processed using Gaussian robust regression model to remove noisy data (32). Processed activity and heart rate data were combined using branched modelling equations to estimate activity intensity time-series which were summarised as daily PAEE of the participants (19, 33). For participants who were not able to complete a step test for individual calibration of their heart rate-energy expenditure relationship, we used a group calibration equation which was derived as the average age and sex-specific calibration curves from the participants who performed the step test (34, 35).

Sleeping heart rate was determined using the highest value of the thirty lowest minute-by-minute heart rate readings during a 24-hours day according to the manufacturer protocol (36) and an average of SHR for all the days was used for analysis.

Prior to free-living monitoring, participants performed a step test for 5-minutes and followed by 2-minutes recovery period for individual calibration of the relationship between heart rate and energy expenditure (34). In addition, heart rate recorded during the step test was used to determine the estimated maximum uptake of oxygen during exercise (VO_2_ max (mLO_2_/kg/min) using Tanaka equation (21, 36). Physical activity and cardiorespiratory fitness markers were the main independent variables.

Venous blood samples were collected, processed and used for HIV-testing, CD4 count, C-reactive protein (CRP), glucose and insulin assessment. HIV-infection was diagnosed using two rapid tests Bioline (SD Bioline Standard Diagnostic Inc, Giheung-gu, Republic of Korea) and Unigold (Unigold Trinity Biotech, Wicklow, Ireland). Discordant samples were tested using ELISA HIV bio kit (11 Vironostika-HIV Ag/Ab Micro Elisa systems Biomerieuxbv, Netherlands). CD4 count (cells/uL) was determined using Partech Cyflow GmbH machine (Munster, Germany). CRP was measured using sandwich ELISA (37).

After 8 hours of overnight fasting, fasting plasma glucose was determined using a point-of-care hemocue machine (Hemocue AB, Angelholm, Sweden). Thereafter, participants underwent an oral glucose tolerance test (OGTT) and were provided with 82.5 g of dextrose monohydrate (equivalent to 75 g of glucose anhydrous) diluted in 250 mls of drinking water to drink within 5 minutes. The OGTT glucose assessment was done at 0hr (fasting), 30 min, and 120 min. Insulin assessment was conducted in Denmark using ELISA technique (ALPCO, Salem, NH, USA).

We computed HOMA-β index, insulinogenic index, overall insulin release index and oral disposition index as markers of β-cell dysfunction, and HOMA-Insulin Resistance (HOMA-IR) and Matsuda index as markers of insulin resistance as previously described (38–41) (**Supplementary Table 1**). Using data from the full CICADA cohort and Liu’s method (42), these markers were dichotomized using optimal cut-off-points to indicate status of β-cell dysfunction and insulin resistance. β-cell dysfunction was defined as insulinogenic index <0.71 (mU/L)/(mg/dL), HOMA-β index<38.3 (mU/L)/(mmol/L), overall insulin release index <33.3 (mU/L)/(mmol/L) and oral disposition index<0.16 (mU/L)/(mg/dL) (mU/L)^-1^ (7, 38, 39, 41, 43). Insulin resistance was HOMA-IR index >1.9 (mU/L)/(mmol/L) or Matsuda index <7.2 (mU/L)/(mmol/L) (39, 40, 43–45). According to WHO guidelines (46), participants whose 2-hours OGTT glucose level was <7.8, and ≥7.8 to <11.1 mmol/L were classified as having no diabetes, and pre-diabetes, respectively, and those with glucose level of ≥11.1 mmol/L were classified as having diabetes. β-cell dysfunction and insulin resistance markers, pre-diabetes and diabetes were the dependent variables.

### Data management and statistics

Data were processed and analysed using Stata version 16 (STATA Corp LLC, College Station, Texas, USA). We tested for normality of all continuous variables using histograms. Log transformation was done to achieve normal distribution for CRP. We tested for collinearity for all of the continuous independent variables using variance inflation factors (VIF); no corrective measures were needed since all variables had VIF <5. We divided the main continuous independent variables (PAEE, SHR, and VO_2_max) by 5 for easier interpretation of the results. The 5 units change of the independent variables was presented as associated with the outcome variables (β-cell dysfunction and insulin resistance markers, prediabetes and diabetes).

Participants’ characteristics were summarised as numbers (percentages), medians (IQR) or means (SD). For comparisons of two groups, we used chi-square test for categorical variables, t-test for continuous variables which were normally distributed, and Mann-Whitney test for continuous variables which were not normally distributed.

We assessed the relationship of PAEE, SHR, and VO_2_ max with β-cell dysfunction and insulin resistance markers using logistic regression while relationships with pre-diabetes and diabetes were assessed using multinomial logistic regression. We first used minimally adjusted models with age and sex included as potential confounders. Final models were further adjusted for other potential confounders (HIV-status, fat mass, fat-free mass index and log-transformed CRP) known to be independently associated with β-cell dysfunction, insulin resistance, pre-diabetes and diabetes (47, 48). To control for the effect of body size on VO_2_ max with an outcome variables (since VO_2_ max and body mass index were negatively correlated), we added height in the model (49). To further explore the data on the relationships between physical activity and cardiorespiratory fitness with β-cell dysfunction and insulin resistance, we conducted secondary analyses where PAEE, SHR, and VO_2_ max were included in models as tertiles (lower, middle and upper) rather than continuous variables and also made scatter plots to assess the relationship between the outcome and exposure variables (**Supplementary Figure 1**). In an additional analysis, we assessed the association of percentage of sedentary time spent with insulin resistance. We also conducted sensitivity analysis to investigate if exclusion of participants with diabetes improves the association of independent variables with insulin resistance markers (HOMA-IR and Matsuda). We tested for interactions between HIV-status and all independent variables. If we identified an interaction, results were reported by HIV-status. The associations were presented as odds ratio (OR) for logistic regression and relative risk ratio (RRR) for multinomial logistic regression with 95% confidence interval (CI) and p-value respectively. A *p*-value <*0.05* indicated statistically significant differences.

### Ethics consideration

This study was conducted in accordance with declaration of Helsinki of 1964. Ethical approval was provided by the Medical Research Coordinating Committee of the National Institute for Medical Research in Tanzania and Catholic University of Health and Allied Sciences Ethics Review Board. All eligible participants were informed of the study purpose, procedures, benefits and risks by trained staff and completed a written informed consent form prior to their enrolment. Participants who were diagnosed with diabetes were referred to the Sekou Toure regional and referral hospital for further investigations and management.

## Results

Participants recruited to the study were 457 PLWH ART- naive and HIV-uninfected individuals. However, after data processing, 66 (14%) participants were removed from the analysis due to missing data on heart rate and acceleration, leaving 391 (86%) participants. Data for 272 PLWH and 119 HIV-uninfected adults were available for analysis. The mean age was 39 ( ± 10.5) years and 60% (n=235) were females, and these did not differ by HIV-status (**Table 1**). The majority of the participants had primary level of education and worked in the informal sector as self-employed/petty traders. Nutritional status categories differed by HIV-status (p<0.01*)*; this was likely due to higher prevalence of overweight (24.4% *vs* 9.2%) but lower prevalence of underweight (5.0% *vs* 26.5%) among HIV-uninfected compared to PLWH. Similar results were seen for abdominal obesity, as more HIV-uninfected were obese than PLWH (24% *vs* 8%, p=0.01). PAEE was higher in HIV-uninfected compared with PLWH (40.7 ± 17.0 kj/kg/day *vs* 33.4 ± 18.8 kj/kg/day, p=0.001), but SHR was lower in HIV-uninfected compared with PLWH (60.8 (7.9) beats/min *vs* 68.5 (12.1) beats/min, p<0.0001). Among the 391 participants, 259 (66%) had completed step test to derive VO_2_ max data for analysis. For those who did the step test there was no difference in VO_2_ max between PLWH and HIV-uninfected individuals (p=0.97*)*. Comparing those who completed the step test and those who did not do step test, there was no difference in HIV status (p=0.26*)*, but there were differences in age and sex (p=0.0002, p=0.02 respectively) (**Supplementary Table 2**).

**Table 1:** Background characteristics of 391 participants included in the study.

	HIV-infected (n=272)	HIV-uninfected (n=119)	*P* values
Age (years), mean (SD)	38.5 (11.0)	39.8 (10.5)	0.27
Female sex, n (%)	163 (60.0)	72 (61.0)	0.92
Weight(kg), mean (SD)	56 (11.6)	65.0 (13.4)	**<0.0001**
Height (m), mean (SD)	1.63 (0.08)	1.63 (0.08)	0.73
Body mass index (kg/m^2^), n (%)			
Normal 18.5 – 24.99 (kg/m^2^)	156 (57.3)	70 (59.0)	**<0.0001**
Underweight <18.5 (kg/m^2^)	72 (26.5)	6 (5.0)	
Overweight >24.99-<30 (kg/m^2^)	25 (9.2)	29 (24.4)	
Obese ≥30 (kg/m^2^)	19 (6.9)	14 (11.8)	
Abdominal obesity^a^, n (%)	22 (8.0)	29 (24.4)	**<0.0001**
CD4 counts (cells/µL), n (%)			
≥500	36 (13.2)	97 (81.5)	**<0.0001**
201-499	100 (36.8)	20 (16.8)	
≤ 200	136 (50.0)	2 (1.7)	
C-reactive protein (mg/L), median (IQR)	3.4 (1.2, 17.1)	1.4 (0.7, 3.4)	**<0.0001**
Physical activity energy expenditure (PAEE) (kj/kg/day)^b^, mean (SD)	33.4 (18.8)	40.7 (17.0)	**<0.0001**
Sleeping heart rate (SHR) (beats/min)^b^, mean (SD)	68.5 (12.1)	60.8 (7.9)	**<0.0001**
Maximum uptake of oxygen during exercise (VO_2_ max) (mLO_2_/kg/min)^b^, mean (SD)	34.1 (6.2)	34.1 (5.9)	0.97
Fasting insulin 0 hours (µUmL) median (IQR)	4.7 (2.9, 7.5)	6.3 (4.2,9.8)	**<0.0001**
Glucose levels at 0 hours ((mmol/L), mean (SD)	6.4 (1.2)	6.5 (1.0)	0.51
Glucose levels at 2 hours ((mmol/L), mean (SD)	8.4 (2.4)	7.9 (2.0)	**0.03**
Insulinogenic index (mU/L)/ (mg/dL)^c^, median (IQR)	0.60 (-1.7, 3.2)	0.47 (-1.9, 3.8)	0.88
<0.71 (mU/L)/ (mmol), n (%)	140 (51.5)	61 (51.3)	0.96
HOMA-β index (mU/L)/ (mmol/L)^c^,median (IQR)	33.4 (21.0, 55.2)	41.5 (28.2, 78.7)	**0.001**
<38.3 (mU/L)/ (mmol), n (%)	156 (57.4)	53 (44.5)	**0.02**
OIS index (pmol/L)/ (mmol/L)^c^, median (IQR)	30.9 (21.4, 44.9)	39.6 (30.2, 59.8)	**<0.0001**
<33.3 (pmol/L)/ (mmol/L), n (%)	131 (48.2)	30 (25.2)	**0.001**
ODI index (mU/L)/(mg/dL)(mU/L)^-1c^, median (IQR)	0.21 (0.12, 0.36)	0.21 (0.11, 0.37)	0.92
<0.16 (mU/L)/(mg/dL)(mU/L)^-1^, n (%)	95 (35.2)	45 (39)	0.54
HOMA-IR index (mU/L)/ (mmol/L)^d^, median (IQR)	1.3 (0.8, 2.2)	1.8 (1.2, 2.6)	**0.0001**
>1.9 (mU/L)/ (mmol/L), n (%)	89 (32.7)	57 (47.9)	**0.004**
Matsuda index (mU/L)/ (mg/dL)^d^, median (IQR)	6.7 (4.6, 10.1)	5.3 (3.6, 7.8)	**<0.0001**
<7.2 (mU/L)/ (mg/dL), n (%)	122 (44.9)	36 (30.3)	**0.01**
Diabetes by oral glucose tolerance test^e^ (mmol/L), n (%)			
Prediabetes	121 (44.5)	48 (40.7)	0.09
Diabetes	23 (8.5)	4 (3.4)	

^a^Cut off points for normal waist circumference (women: ≤88 cm; men: ≤102 cm) and abdominal obesity (women: >88 cm; men: >102 cm), ^b^256 participants had step test data for VO2 max, 391 had participants PAEE and SHR data, ^c^Markers for β-cell dysfunction ^d^Markers for insulin resistance, ^e^Defined as normal<7.8mmol/L, pre-diabetes≥7.8 -11.1mmol/L, and diabetes>11.1mmol/L. N= number, SD=standard deviation, IQR= inter-quartile range, HOMA-β=Homoeostatic model assessment for β-cell function, HOMA-IR=homoeostatic model of assessment for insulin resistance. Bold p-values represent significant result.

Fasting insulin was lower in PLWH compared to HIV-uninfected individuals (4.7µuml/L vs 6.3µuml/L), but there was no difference in fasting glucose level between these groups (6.4 ± 1.2 mmol/L *vs* 6.5 ± 1.0 mmol/L, p=0.51*).* The OGTT glucose level at 2 hours was higher in PLWH compared with HIV-uninfected individuals (8.4 ± 2.4 mmol/L *vs* 7.9 ± 2.1 mmol/L, p=0.03*).* PLWH had lower median HOMA-β compared with HIV-uninfected individuals (33.4 (mU/L)/(mmol/L) *vs* 41.5, (mU/L)/(mmol/L), p=0.01*).* Similarly, PLWH had lower median HOMA-IR compared with HIV-uninfected individuals (1.3 (mU/L)/(mmol/L) *vs* 1.8, (mU/L)/(mmol/L), p=0.004*)*. There were higher percentages of β-cell dysfunction as defined by HOMA-β index and overall insulin release index among PLWH compared with HIV-uninfected adults ((57.4% *vs* 44.5%, p=0.02) and (48.2% *vs* 25.2%, p=0.001), respectively). The percentage of people with insulin resistance as defined by HOMA-IR was lower among PLWH compared with HIV-uninfected adults (32.7% vs 47.9%, p=0.004) whereas when defined by Matsuda index insulin resistance was higher among PLWH compared with HIV-uninfected adults (44.9% vs 30.3%, p=0.01*)* (**Table 1**).

**Tables 2**–**5** present the associations between PAEE, SHR and VO2 max with β-cell dysfunction and insulin resistance markers, prediabetes and diabetes. HIV-status did not modify the associations of physical activity and cardiorespiratory fitness markers on β-cell dysfunction and insulin resistance markers, prediabetes and diabetes (p for interaction >0.05, all independent variables). Therefore, results on these associations presented below are not stratified by HIV-status. In sensitivity analysis, we found that exclusion of participants with diabetes did not improve the association of independent variables with insulin resistance markers (**Supplementary Table 3**)

**Table 2:** The association of physical activity energy expenditure, sleeping heart rate and maximum uptake of oxygen during exercise with β-cell dysfunction and insulin resistance among people living with HIV and HIV-uninfected adults .

	Model 1^a^ (minimally adjusted)						Model 2^b^ (fully adjusted)					
	PAEE^c^(n=391)		SHR^d^(n=391)		VO_2_ max^e^(n=259)		PAEE^c^(n=391)		SHR^d^(n=391)		VO_2_ max^e^N=259	
	OR (95% CI)	*P value*	OR (95% CI)	*P value*	OR (95% CI)	*P value*	OR (95% CI)	*P value*	OR (95% CI)	*P value*	OR (95% CI)	*P value*
**β-cell dysfunction markers**												
Insulinogenic index<0.71(mU/L)/ (mg/dL)	0.94 (0.89, 0.99)	**0.04**	1.02 (0.93, 1.11)	0.67	0.86 (0.69, 1.08)	0.21	0.95(0.89,1.02)	0.16	0.99 (0.89, 1.11)	0.99	0.96 (0.75, 1.24)	0.77
Oral disposition index<0.16(mU/L)/(mg/dL)(mU/L)^-1^	0.93 (0.87,0.99)	**0.02**	1.06 (0.96, 1.16)	0.22	0.78 (0.61, 1.01)	0.06	0.95 (0.89, 1.02)	0.17	1.04 (0.93, 1.16)	0.48	0.92 (0.69, 1.21)	0.55
HOMA-β^f,^ <38.3 (mU/L)/(mmol/L)	1.01 (0.95, 1.06)	0.80	1.14 (1.03, 1.25)	**0.01**	1.20 (0.95, 1.51)	0.11	1.03 (0.97, 1.11)	0.31	1.05 (0.92, 1.18)	0.48	1.95 (0.71, 1.27)	0.72
Overall insulin release index^h^<33.3 (pmol/L)/ (mmol/L)	1.01 (0.99, 1.06)	0.84	1.11 (1.01, 1.22)	**0.02**	1.29 (1.02, 1.63)	0.03	1.04 (0.97, 1.11)	0.25	1.02 (0.91, 1.13)	0.73	1.12 (0.85, 1.46)	0.18
**Insulin resistance markers**												
HOMA-IR^g,^ >1.9 (mU/L)/(mmol/L)	0.94 (0.88, 1.01)	**0.05**	0.92 (0.83, 1.02)	0.11	0.74 (0.58, 0.95)	**0.02**	0.91 (0.84, 0.98)	**0.01**	1.00 (0.88, 1.13)	0.97	0.94 (0.69, 1.26)	0.67
Matsuda index^i^<7.2 (mU/L)/ (mg/dL)	0.96 (0.90, 1.02)	0.18	0.90 (0.82, 0.99)	**0.03**	0.78 (0.62, 1.00)	**0.05**	1.07 (1.00, 1.14	**0.04**	1.02 (0.90, 1.14)	0.78	1.07 (0.81, 1.42)	0.62

^a^Model 1 adjusted for age and sex, ^b^Model 2, adjusted for age, sex, HIV-status, fat mass/fat-free mass index and log-transformed C-reactive protein ^c^PAEE=physical activity energy expenditure (kj/kg/day), ^d^SHR=sleeping heart rate (beats/min), ^e^VO2 max=maximum uptake of oxygen during exercise (mLO2/kg/min),^f^HOMA-β=Homoeostatic model assessment for β-cell function, ^g^HOMA-IR=homoeostatic model of assessment for insulin resistance OR=Odds ratio, CI=Confidence interval. Bold p-values represent significant results

**Table 3:** Secondary analysis of the association of physical activity energy expenditure, sleeping heart rate and maximum uptake of oxygen during exercise with β-cell dysfunction among people living with HIV and HIV-uninfected adults.

Lower insulinogenic index ^a^ <0.7(mU/L)/(mg/dL)					Lower HOMA-β index ^a,b^ <38.3 (mU/L)/(mmol/L)				Lower overall insulin release index ^a^<33.3 (pmol/L)/(mmol/L)				Lower oral disposition index^a^<0.16 (mU/L)/(mg/dL) (mU/L)^-1^			
	Model 1^c^ (minimally adjusted)		Model 2^d^ (fully adjusted)		Model 1^c^ (minimally adjusted)		Model 2^d^ (fully adjusted)		Model 1^c^ (minimally adjusted)		Model 2^d^ (fully adjusted)		Model 1 (minimally adjusted)		Model 2 (fully adjusted )	
	OR (95%CI)	*p value*	OR (95%CI)	*p value*	OR (95%CI)	*p value*	OR (95%CI)	*P value*	OR (95%CI)	*p value*	OR (95%CI)	*P value*	OR (95CI)	*P value*	OR (95CI)	*P value*
Physical activity energyexpenditure^f^ (kj/kg/min)^f^																
Lower tertile	Ref		Ref		Ref		Ref		Ref		Ref		Ref		Ref	
Middle tertile	0.52 (0.32, 0.85)	**0.01**	0.48 (0.27, 0.82)	**0.01**	0.63 (0.38, 1.05)	0.07	0.97 (0.53, 1.80)	0.93	0.50 (0.30, 0.84)	**0.01**	0.67 (0.38, 1.19)	0.17	0.83 (0.50, 1.39)	0.48	0.89 (0.51, 1.58)	0.71
Upper tertile	0.57 (0.35, 0 95)	**0.02**	0.63 (0.36, 1.11)	0.11	0.88 (0.53, 1.47)	0.63	1.02 (0.55 ,1.91)	0.93	0.78 (0.47, 1.30)	0.34	0.97 (0.54, 1.72)	0.91	0.57 (0.33, 0.97)	**0.04**	0.73 (0.40, 1.31)	0.29
Sleeping heart rate^f^(beats/min)																
Lower tertile	Ref		Ref		Ref		Ref		Ref		Ref		Ref		Ref	
Middle tertile	0.74 (0.46, 1.21)	0.24	0.76 (0.46, 1.27)	0.30	1.24 (0.75, 2.04)	0.41	1.09 (0.62, 1.94)	0.76	0.97 (0.59, 1.61)	0.91	0.86 (0.50, 1.49)	0.60	1.51 (0.90, 2.54)	0.12	1.54 (0.89, 2.64)	0.12
Upper tertile	0.97 (0.58, 1.63)	0.92	0.93(0.50, 1.70)	0.81	1.99 (1.15, 3.43)	**0.01**	1.22 (0.62, 2.41)	0.57	1.96 (1.15, 3.34)	**0.01**	1.22 (0.65, 2.29)	0.53	1.16 (0.66, 2.01)	0.61	0.99 (0.52, 1.88)	0.97
VO_2_ max^e f^(mLO_2_/kg/min)																
Lower tertile	Ref		Ref		Ref		Ref		Ref		Ref		Ref		Ref	
Middle tertile	0.63 (0.34, 1.16)	0.14	0.78 (0.41, 1.51)	0.46	1.52 (0.82, 2.83)	0.18	0.84 (0.39, 1.78)	0.64	0.74 (0.38, 1.41)	0.14	0.46 (0.22, 0.94)	**0.04**	0.78 (0.43, 1.49)	0.46	1.21 (0.56, 2.28)	0.74
Upper tertile	1.09 (0.57, 2.09)	0.79	1.42 (0.69, 2.93)	0.34	1.36 (0.70, 2.63)	0.37	0.83 (0.37, 1.90)	0.67	1.91 (0.97, 3.77)	0.79	1.28 (0.60, 2.76)	0.52	0.72 (0.35, 1.47)	0.37	1.03 (0.47, 2.28)	0.93

^a^lower insulinogenic index, lower HOMA-β index, lower overall insulin release index and lower oral disposition index are markers of β-cell dysfunction. ^b^HOMA-β=Homoeostatic model assessment for β-cell function, ^c^Model 1 adjusted for age and sex, ^d^Model 2 adjusted for age, sex, HIV-status, fat mass,/fat-free mass index and log-transformed C-reactive protein ^e^VO2 max=maximum uptake of oxygen during exercise (mLO2/kg/min), OR= odds ratio, CI=confidence interval. ^f^391 participants have been assessed PAEE and SHR and 259 participants have been assessed participants. Bold p-values represent significant results

**Table 4:** Secondary analysis of the association of physical activity energy expenditure, sleeping heart rate and maximum uptake of oxygen during exercise with insulin resistance among people living with HIV and HIV-uninfected adults .

	Lower Masuda Index ^a^<7.2 (mU/L)/(mg/dL)				Higher HOMA-IR ^a,b^>1.9 (mU/L)/(mmol/L)			
	Model 1^c^ (minimally adjusted)		Model 2^d^ (fully adjusted)		Model 1^c^ (minimally adjusted)		Model 2^d^ (fully adjusted)	
	OR (95%CI)	*p value*	OR (95%CI)	*p value*	OR (95%CI)	*p value*	OR (95%CI)	*p value*
Physical activity energy expenditure (kj/kg/day)^f^								
Lower tertile	Ref		Ref		Ref		Ref	
Middle tertile	1,27 (0.75, 2.15.31)	0.35	1.31 (0.71, 2.42)	0.39	1.43 (0.85, 2.39)	0.18	0.99 (0.53,1.83)-	0.97
Upper tertile	0.70 (0.42, 1.18.37)	0.18	1.95 (1.06,, 3.60)	**0.03**	0.65 (0.37, 1.12)	0.12	0.52 (0.27, 0.99)-	**0.05**
Sleeping heart rate(beats/min)^f^								
Lower tertile	Ref		Ref		Ref		Ref	
Middle.tertile	1.10 (0.66, 1.84)	0.71	0.74 (0.42, 1.30)	0.30	0.92 (0.55, 1.55)	0.76	1.06 (0.60, 1.91)-	0.82-
Upper tertile	0.58 (0.34, 1.01)	**0.05**	1.04 (0.54, 2.00)	0.91	0.59 (0.34, 1.05)	0.07	0.93 (0.46, 1.86)	0.84
VO_2_ max^ef^ (mLO_2_/kg/min				–				
Lower tertile	Ref		Ref	Ref	Ref		Ref	
Middle tertile	0.83 (0.42, 1.62)	0.59	0.77 (0.37, 1.63)	0.50	0.5 (0.27, 0.97)	**0.04**	0.87 (0.41, 1.84)	0.72
Upper tertile	0.54 (0.27, 1.09)	0.08	1.25 (0.56, 2.79)	0.58	0.53 (0.26, 1.06)	0.07	0.81(0.36, 1.84)	0.62

^a^Lower Matsuda index, higher HOMA-IR index are markers of insulin resistance. ^b^HOMA-IR=Homoeostatic model assessment for insulin resistance, ^c^Model 1 adjusted for age and sex, ^d^Model 2 adjusted for age, sex, HIV-status, fat/fat-free mass index and log-transformed C-reactive protein ^e^VO2 max=maximum uptake of oxygen during exercise (mLO2/kg/min), OR= odds ratio, CI=confidence interval. ^f^391 participants have been assessed PAEE and SHR, 259 participants have been assessed VO2 max. Bold p-values represent significant results

**Table 5:** The association of physical activity energy expenditure, sleeping heart rate and maximum uptake of oxygen during exercise with prediabetes and diabetes among people living with HIV and HIV-uninfected adults .

			Model 1^a^ (minimally adjusted)						Model 2^b^ (fully adjusted)			
	PAEE^c^(n=391)		SHR^d^(n=391)		VO2 max^e^(n=259)		PAEE^c^(n=391)		SHR^d^N=391		VO2 max^e^N=259	
	RRR (95% CI)	*P value*	RRR (95% CI)	*P value*	RRR (95% CI)	*P value*	RRR (95% CI)	*P value*	RRR (95% CI)	*P value*	RRR (95% CI)	*P value*
Prediabetes^f^												
	0.84 (079, 0.90)	**<0.001**	1.18 (1.06, 1.31)	**0.003**	0.89 (0.85, 0.95)	**<0.001**	0.98 (0.96, 0.99)	**0.001**	1.02 (0.99, 1.05)	0.07	0.91 (0.86, 0.97)	**0.001**
Diabetes^g^	0.56 (0.45, 0.69)	**<0.001**	1.63 (1.36, 1.94)	**<0.001**	0.85 (0.75, 0.96)	**0.01**	0.92 (0.88, 0.96)	**<0.001**	1.06 (1.01, 1.11)	**0.005**	0.92 (0.80, 1.06)	0.27

^a^Model 1 adjusted for age and sex, ^b^Model 2 adjusted for age, sex, HIV-status, fat mass/fat-free mass index, and log-transformed C-reactive protein. ^c^PAEE=physical activity energy expenditure (kj/kg/day),^d^SHR=sleeping heart rate (beats/min), ^e^VO2 max=maximum uptake of oxygen during exercise (mLO2/kg/min) ^f^Defined as two-hour oral glucose tolerance testglucose ≥7.8 -11.1 mmol/L, ^g^Defined as two-hour oral glucose tolerance testglucose ≥11.1mmol/L. RRR= relative risk ratio, CI= confidence interval. Bold p-values represent significant results.

In the fully adjusted models, an increment of 5 kj/kg/day of PAEE was not associated with lower odds of abnormal insulinogenic index, HOMA-β and overall insulin release index (**Table 2**). When PAEE was analysed as tertiles, we found that compared to the lowest tertile, the middle tertile was associated with lower odds of abnormal insulinogenic index (OR=0.48, 95%CI: 0.27, 0.82) (**Table 3**). We also found in minimally adjusted model, compared to lower tertile, upper tertile of PAEE was associated with lower odds of abnormal oral disposition index (OR=0.57,95%CI: 0.33, 0.97) but this was not significant in a fully adjusted model (**Table 3**). SHR and VO_2_ max were not associated with insulinogenic index, HOMA-β, overall insulin release index or oral disposition index in fully adjusted models in either the main or the secondary analyses except for VO_2_ max, compared to low tertiles, middle tertile was associated with lower odds of overall insulin release index (**Tables 2**, **3**).

With insulin resistance, an increase of every 5 kj/kg/day of PAEE was associated with lower odds of abnormal HOMA-IR (OR=0.91, 95%CI: 0.84, 0.98) and higher odds of higher Matsuda index (OR=1.07, 95%CI: 1.00, 1.14) (**Table 2**), this was similar in the secondary analysis using categorical variables for upper tertiles (**Table 4**). In an additional analysis, high percentage of time spent on sedentary activities was associated with higher odds of abnormal HOMA-IR (OR=38.38, 95%CI: 2.78, 528.59) (**Supplementary Table 4**). SHR and VO_2_ max were not associated with HOMA-IR or Matsuda index in the main and secondary analyses (**Table 2**, **Table 4**).

In the fully adjusted models which included age, sex, HIV-status, fat mass and fat free mass and CRP, a change of 5 kj/kg/day of PAEE was associated with reduced risk of pre-diabetes (RRR=0.98, 95%CI: 0.96, 0.99) and diabetes (RRR=0.92, 95%CI: 0.88, 0.96) while an increment of 5 beats/min of SHR was associated higher risk of diabetes (RRR=1.06, 95%CI: 1.01, 1.11) (**Table 5**). An increment of 5 mLO_2_/kg/min of VO_2_ max was associated with lower risk of pre-diabetes (RRR=0.91, 95%CI: 0.86, 0.97) but not diabetes.

## Discussion

In this study, we assessed the association of objectively measured habitual physical activity and cardiorespiratory fitness with β-cell dysfunction, insulin resistance and diabetes among ART-naïve PLWH and HIV-uninfected adults. Physical activity but not cardiorespiratory fitness was associated with lower risk of β-cell dysfunction and insulin resistance. Physical activity was associated with lower risk of prediabetes and diabetes. Cardiorespiratory fitness indicated by VO_2_ max was associated with lower risk of prediabetes, but not diabetes, and higher SHR was associated with higher risk of diabetes, but not prediabetes.

In this analysis, we found that the middle tertile of PAEE was associated with lower risk of β-cell dysfunction (defined by insulinogenic index) and the direction of the association did not change when using oral disposition index which is a better marker of β-cell function because it controls for insulin sensitivity. This indicates that participants who were involved in longer habitual moderate to high intensity physical activity may have had increased insulin secretion. These findings are similar to other studies from high-income countries (50). Most of those studies which were conducted in supervised intensified physical activity programs rather than free-living environment have reported that the effect of physical activity on β-cell dysfunction depends on the intensity and duration of physical activity (51, 52). The relationship of PAEE and reduced risks of β-cell dysfunction could be explained by several mechanisms which include intracellular molecular activities that are important for increasing β-cell mass and β-cell insulin secretion (50–52). High intensity physical activity allows secretion of interleukin-6 in the muscles which leads to an increase in the levels of circulating glucagon-like peptide which may protect β-cells from apoptosis and promote cell growth (51, 52).

Our study assessed the association between VO_2_ max and β-cell dysfunction. Previous studies have reported high intensity physical activity and VO_2_ max was associated with better β-cell function and glycaemic control (24, 53). Unlike these studies indicating high VO_2_ max was protective factor of β-cell dysfunction; we did not find that association in this population. We would have expected that, like PAEE, VO_2_ max to be associated with reduced risk of β-cell dysfunction because of the dependance on each other. High level of PAEE correlates with high levels of VO_2_ max. Nevertheless, the results we observed could be explained by our participants using a step test rather than the walking tests in measuring VO_2_ max (54). In addition, our participants could have been genetically different from those in published studies. Thus, this may have led to the differences since there is evidence that VO_2_ max is partly influenced by genetics (53). In view of these results and the fact that there are few studies in the SSA assessing cardiorespiratory fitness in adult population, and its association with β-cell dysfunction (55) there is a need for further research to assess the relationship between cardiorespiratory fitness and β-cell function in African populations.

Insulin resistance may be caused by genetic abnormalities as well as increased visceral adiposity and overweight (56). However, moderate intensity physical activity including simple interval walking activities can increase insulin uptake in the muscles leading to higher insulin sensitivity (57, 58) and lower risk of diabetes (59). Our study reports similar findings to those reported in other settings on the association between physical activity and insulin resistance (60, 61). Also, results of this study are similar to other studies using objective physical activity measures. A study in Australia which investigated the association of physical activity and cardio-metabolic risk factors using the International Physical Activity Questionnaire (IPAQ) and pedometers (equipment for objectively measuring physical activity), found that the objective measures of physical activity were more consistently associated with adverse cardiometabolic risk factors than were subjective IPAQ results (62). Subjective measures of physical activity are subject to under- or over-reporting which could underestimate or overestimate associations with health outcomes (62). Our study used PAEE, a measure validated against doubly labeled water method, a gold standard for assessing habitual energy expenditure (15). Thus, our results contribute to existing evidence suggesting objectively measured habitual physical activity is associated with improved glucose metabolism.

We found that physical activity was associated with lower risk of prediabetes and diabetes. This association is probably mediated by the positive effects of physical activity on both β-cell function and insulin resistance. These results suggest that promotion of physical activity interventions is needed to prevent the rising burden of diabetes in SSA (63, 64). In addition, physical activity could delay the progression of non-insulin dependent diabetes to insulin-dependent diabetes (65), but further research is needed to investigate this potential beneficial effect of physical activity.

High VO_2_ max and low SHR have been reported to reduce the risk of insulin resistance and diabetes (66, 67). In our study, we found no association between SHR and VO_2_ max with insulin resistance but found that higher SHR was associated with higher risk of diabetes and VO_2_ max was associated with lower risk of prediabetes (but not diabetes). Our participants had an average VO_2_ max of 34 mLO_2/_kg/min which is lower than the average reported in other African studies (24), and seemed to have a higher SHR compared to other populations (68). These differences in population characteristics could explain the difference between our results and those reported in other studies.

The diabetes risk is higher among ART-naïve PLWH compared to HIV-uninfected adults. This may be partly because PLWH have low levels of physical activity. In contrast to other studies (69), we did not find that HIV modifies the effect of physical activity and cardiorespiratory fitness on β-cell dysfunction, insulin resistance and diabetes.

## Strengths and limitations

The strengths of this study are the use of objective measures to assess habitual physical activity and cardiorespiratory fitness using PAEE as well as SHR and VO_2_ max, inclusion of both PLWH and HIV-uninfected participants, and an adequate sample size to quantify the associations of independent variables with β-cell function, insulin resistance, pre-diabetes and diabetes in the SSA. Additionally, we used individual calibration of heart rate *via* a step test in the majority of the participants and a population-specific group calibration equation for estimating PAEE in those who did not complete a step test; this approach limits the bias stemming from using calibration equations from populations with a different heart rate response (35).

Our study used the estimated VO_2_ max computed from a 5-minute step test which is not a gold standard test. We acknowledge the differences in participants’ characteristics of those who did the step test and those who did not do the step test. This may have reduced the generalisability of our findings to the general population and we did not account for multiple testing of our outcomes.

We have used oral glucose tolerance test as a diagnostic test for diabetes, this may have underestimated diabetes prevalence. This study was cross-sectional, limiting causal inference between physical activity and cardiorespiratory fitness with β-cell function, insulin resistance and pre-diabetes/diabetes. Longitudinal studies are needed to confirm the observed associations and to investigate the mechanisms explaining associations among PAEE, SHR, and VO_2_ max with β- cell function and insulin resistance among African adults.

## Conclusions

As we found low physical activity to be associated with β-cell dysfunction and insulin resistance, physical activity should be promoted to prevent diabetes in SSA among PLWH and HIV-uninfected individuals. Future studies using randomised control trial design would be useful for evaluating physical activity to prevent diabetes and delay progression of non-insulin dependent to insulin-dependent diabetes in PLWH and HIV-uninfected populations in SSA.

## Data availability statement

The datasets presented in this article are not readily available because data may be requested and approved by the chairperson of the medical research coordinating committee of the National Institute for Medical Research. Requests to access the datasets should be directed to ethics@nimr.or.tz.

## Ethics statement

The studies involving human participants were reviewed and approved by medical research coordinating committee of the National Institute for Medical Research in Tanzania and Catholic University of Health and Allied Sciences Ethics Review Board. The patients/participants provided their written informed consent to participate in this study.

## Author contributions

BK, GP, SF, JC, HF, KJ, RK-M, MO, and DF-J conceived and designed the study. BK, BBK, GP, and KJ collected data. BK analysed the data with support from SB, RP, MFO, DF-J and drafted the paper. All authors interpreted the results, reviewed and approved the final version of the manuscript.

## Funding

This study was funded by the Ministry of Foreign Affairs of Denmark and administered by Danida Fellowship Centre (grant: 16-P01-TAN). RK-M is funded by the Centre for Physical Activity Research (CFAS) and supported by TrygFonden (grants ID 101390 and ID 20045). SB is funded by the UK Medical Research Council (MCUU12015/3) and the NIHR Biomedical Research Centre in Cambridge (IS-BRC-1215–20014). BK is supported by a grant from the Fogarty International Centre of the National Institutes of Health under award number D43TW011295. The funding agencies had no role in the study design, data collection and analysis, preparation of the manuscript, and decision to publish results.

## Acknowledgments

The authors thank all participants for participating in this study. We are grateful to the staff of the CICADA clinic, ART clinics in Mwanza and NIMR laboratory team for their support.

## Conflict of interest

The authors declare that the research was conducted in the absence of any commercial or financial relationships that could be construed as a potential conflict of interest.

## Publisher’s note

All claims expressed in this article are solely those of the authors and do not necessarily represent those of their affiliated organizations, or those of the publisher, the editors and the reviewers. Any product that may be evaluated in this article, or claim that may be made by its manufacturer, is not guaranteed or endorsed by the publisher.
