# Supplementary material for: The association of physical activity and cardiorespiratory fitness with β-cell dysfunction, insulin resistance, and diabetes among adults in north-western Tanzania: A cross-sectional study
Source: Front Endocrinol (Lausanne). 2022 Aug 3;13:885988. doi: 10.3389/fendo.2022.885988 (PMC9381963; doi:10.3389/fendo.2022.885988)

Supplementary Table 1: Formulae for β-cell function and insulin resistance markers

Marker	Definition/formula	Units	References
**β-cell function**			
Insulinogenic index	Change in insulin over change in glucose in first 30 minutes following OGTT.	(mU/L/mg/dL)	[1]
Overall insulin release index	Ratio of AUC of insulin to AUC of glucose from 0 to 120 minutes of OGTT	(pmol/L/mmol/L)	[1, 2]
HOMA-β	(20* Fasting blood insulin (FBI)/(Fasting plasma glucose (FPG)-3.5)	(mU/L, mmol/L)	[1]
Oral disposition index	Insulinogenic index/1/fasting insulin	(mU/L,/ (mgdL) $(mU/L)^{-1}$	[3]
**Insulin resistance**			
HOMA-IR	(FBI *FPG)/22.5	(mU/L, mmol/L)	[1]
Matsuda index	1000/√FPG*FBI) (MPG)*(MPI)	(mU/L, mg/dL)	[1]

AUC, area under the curve; HOMA-β, Homeostatic model assessment-β; HOMA-IR, HOMA-Insulin Resistance; OGTT, Oral glucose tolerance test; MPG, mean plasma glucose at 0, 30 and 120 minutes; MPI, mean of plasma insulin at 0, 30, and 120 minutes.

1. Albareda, M., et al., *Assessment of insulin sensitivity and beta-cell function from measurements in the fasting state and during an oral glucose tolerance test.* Diabetologia, 2000. **43**(12): p. 1507-11.
2. Stancáková, A., et al., *Changes in insulin sensitivity and insulin release in relation to glycemia and glucose tolerance in 6,414 Finnish men.* Diabetes, 2009. **58**(5): p. 1212-21.
3. Utzschneider, K.M., et al., *Oral disposition index predicts the development of future diabetes above and beyond fasting and 2-h glucose levels.* Diabetes Care, 2009. **32**(2): p. 335-41.

Supplementary Table 2: Background characteristics of participants with VO_2 max and without VO_2 max data

	With VO2 Max (N=239)	Without VO2 max (N=132)	p-value
Age (yrs), mean (SD)	37 (10.0)	42 (12.0)	**0.0002**
Sex, female, n (%)	155 (65.9)	80 (34.0)	**0.02**
HIV status			
Negative, n (%)	81 (68.1)	38 (31.9)	0.26
Positive, n (%)	178 (65.4)	94 (34.6)	
Body mass index groups, n(%)			
Normal (18.5–24.99 kg/m^2)	160 (71)	66 (29.)	0.06
Underweight (<18.5 kg/m^2)	42 (54)	36 (46.2)	
Overweight (≥24.99-<30 kg/m^2)	36 (67)	18 (33)	
Obesity(≥30kg/m^2)	21 (64)	12 (36)	

T-test used to compare means for continuous variables and chi square test used to compare percentages for categorical variables.

N= number, SD standard deviation

Supplementary Table 3: The association of physical activity energy expenditure and β-cell dysfunction and insulin resistance among participants who have normalglycemia and pre-diabetes

	Model 1[a](Minimally adjusted)		Model 2[b] (Fully adjusted)	
	PAEE (kj/kg/day) (N=355)		PAEE(kj/kg/day) (N=355)	
Higher HOMA-IR[c](>1.9 (mU/L)/ (mmol/L)	0.96 (0.90, 1.02)	0.23	0.93 (0.86, 1.00)	**0.05**
Lower Matsuda index[c] (<7.2 (mU/L)/ (mg/dL)	1.01 (0.96, 1.08)	0.64	1.05 (0.98, 1.13)	0.16

[a]Model 1 adjusted for age and sex, [b]Model 2 adjusted for age, sex, HIV-status, fat/fat-free mass index and log-transformed C-reactive protein
[c]Lower insulinogenic index, lower HOMA-β, Lower overall insulin release index are markers of β-cell dysfunction, Lower Matsuda index, higher HOMA-IR index are markers of insulin resistance.

Supplementary Table 4: The association of sedentary percent time and insulin resistance among HIV-infected and HIV-uninfected adults.

	Model 1[a] (Minimally adjusted)		Model 2[b] (Fully adjusted)	
	PAEE (kj/kg/day) N=391		PAEE(kj/kg/day) N=391	
	OR (95% CI)	*P value*	OR (95% CI)	*P value*
Higher HOMA-IR[c] (>1.9 (mU/L)/(mmol	6.29 (0.74, 53.34)	0.09	38.38 (2.78, 528.59)	**0.006**
Lower Matsuda index[c] (<7.2 (mU/L)/(mmol)	0.51 (0.06, 4.02)	0.52	0.11 (0.009, 1.22)	0.07

[a]Model 1 adjusted for age and sex, [b]Model 2 adjusted for age, sex, HIV-status, fat/fat-free mass index and log-transformed C-reactive protein
[c]Lower Matsuda index, and higher HOMA-IR index are markers of insulin resistance.

Supplementary figure 1A showing the relationship between physical activity energy expenditure and HOMA_IR

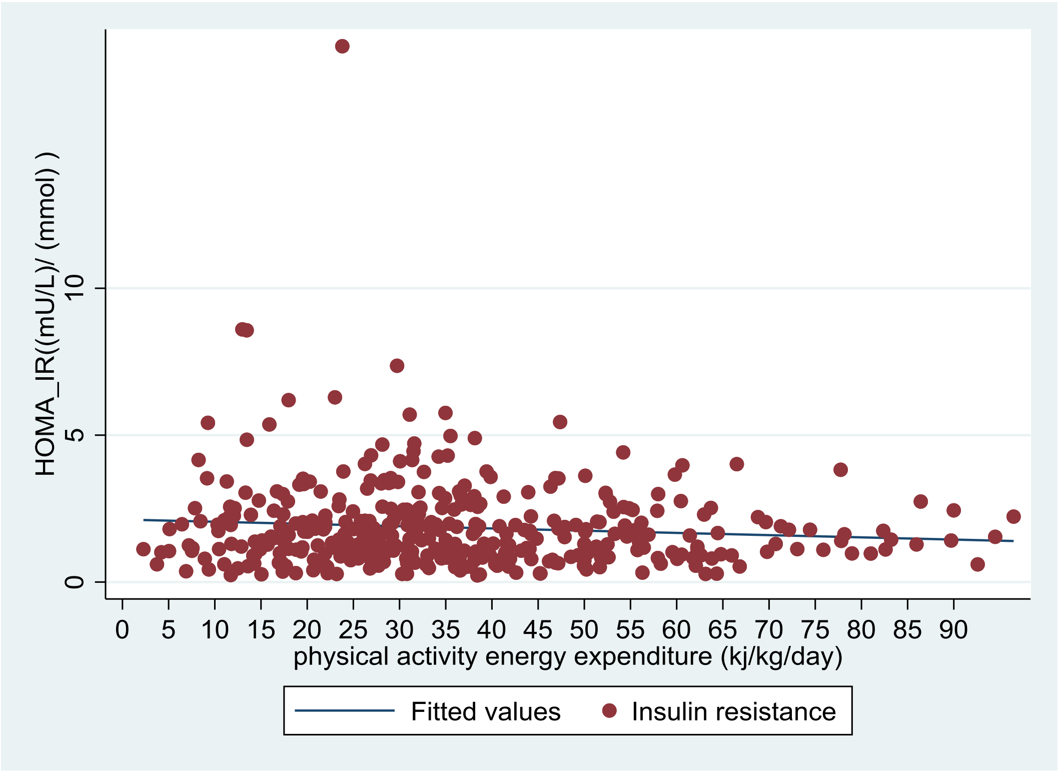

Supplementary figure 1B showing the relationship between physical activity energy expenditure and Matsuda

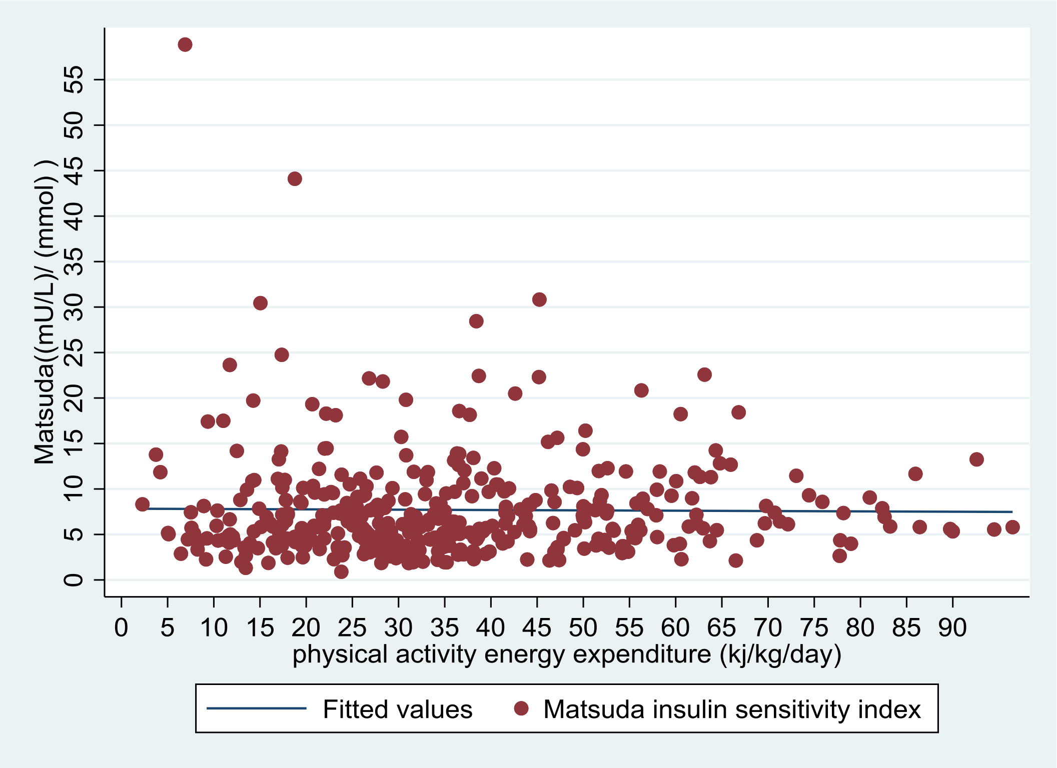

Supplement: Supplementary file 1 [file DataSheet_1.pdf]
